# Low vision care: who can help?

**Published:** 2012

**Authors:** Karin van Dijk

**Affiliations:** CBM global advisor on low vision; low vision consultant to Light for the World Netherlands and to Kilimanjaro Centre for Community Ophthalmology. kvdijknl@yahoo.com

**Figure:**
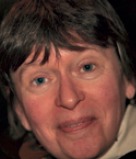
Karin van Dijk

We know that, in many low- and middle-income countries, low vision services are limited to tertiary or teaching hospitals, which means that most people are not able to access them.

If this is the case, who can those with low vision turn to for help?

People with low vision do not fit comfortably within the job descriptions of most health and education professionals.

- They are not blind, so rehabilitation workers may not feel able to help them
- Clinicians (ophthalmologists, ophthalmic nurses, and other mid-level personnel) feel there is nothing more they can do
- Optometrists and refractionists can improve their vision, but cannot help them to see ‘normally’
- Special education teachers are usually trained just to work with children who are blind, and may not have the additional training needed to help children use low vision devices and advise them about where to sit and the importance of using their vision.

In fact, the services of all of these people are vital to ensure that the person with low vision can live a full life.

**Figure:**
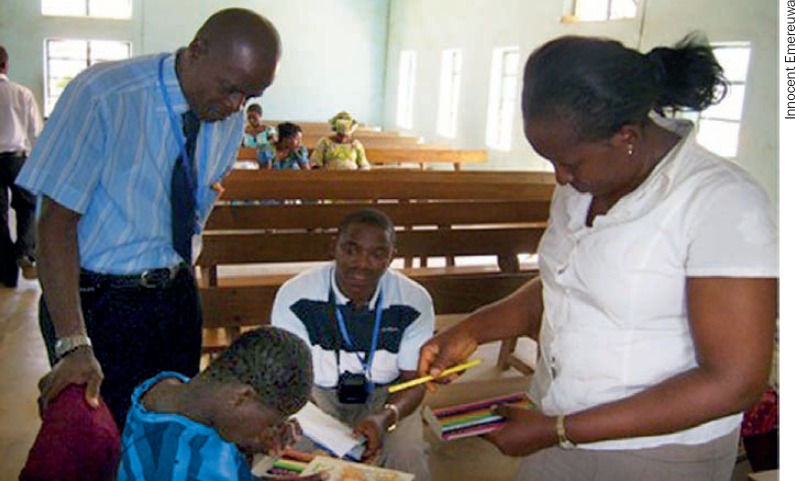
An older child's colour vision is tested during an outreach clinic. NIGERIA

One of the most important things we can do, whatever our own role, is to be aware of what other services may help the person with low vision and refer them. And we must communicate with the person, the family, and our colleagues in these other services about the care the person needs, in clear and simple language.

## Importance of referral

People with low vision may need clinical care, refraction, and rehabilitation support, and children and others in full-time education will also require educational support. We may be the first point of contact for the person with low vision, or their last hope for help. Whatever the case, it is our responsibility to find out whether the people who come to us have received clinical and refractive error care. If they have not, it is essential that we refer them. If they have, we must find out what other support they might need and refer them.

But it is not enough to just refer – it is also our responsibility to make contact with our colleagues in local community rehabilitation and educational support services. Refer people as appropriate, and share information with these colleagues about any changes in the needs and vision-related abilities of the person with low vision.

## Different levels of low vision care

### Primary/community level

Nurses, ophthalmic nurses, community-based workers, and other mid-level personnel can do the following:

- Be alert and identify people who might have low vision
- Refer them for diagnosis, prognosis, and good refraction
- Refer older children and adults who have useful vision to low vision services at secondary or district level
- Refer young children and adults with complex needs to tertiary level
- After diagnosis, refraction, and referral for low vision care, advise on non-optical interventions and environmental modifications (pages 7, 8, and 12) and refer for educational support and community-based rehabilitation if needed.

### Secondary or district level

At secondary or district level, services are aimed mainly at adults and older children who want to access print or perform tasks that require good near vision. The panel on page 14 lists the minimum equipment you will need to start a low vision service at secondary or district level.

At this level, optometrists and mid-level eye care workers can be trained to give basic low vision services appropriate to their skills and experience.

They should have good communication skills and be able to do the following:

- Test distance and near visual acuity (ideally also in younger children)
- Perform objective and subjective refraction
- Perform minimum essential low vision assessments (page 4 onwards)
- Prescribe essential low to medium magnification devices for near and distance, with training in their use (pages 9–10)
- Advise patients on non-optical interventions and environmental modifications (page 12)
- Refer people to the most appropriate person or organisation for further training, financial help, and education
- Refer young children and those with complex needs to the tertiary level
- Ensure regular follow-up of adults and children who were seen at tertiary level.

### Tertiary level or teaching hospital

Well-trained, dedicated low vision staff can provide the following:

- Complex assessment tests
- Refraction of people with complex problems
- Provision of a wide range of devices, including electronic devices
- Good links to education and rehabilitation services
- Training the use of low vision devices.

## Beyond the clinic

There will be many more people with low vision in the community who need our services.

Think about how you can reach out to tell them about what you offer. Plan outreach clinics, or link with others working in the community.

Visit schools for the blind – perhaps there are children who will be able to use their remaining vision if they receive low vision support.

Low vision work may be challenging, but it is immensely rewarding!

**Providing a basic low vision service at district level: what is the minimum we need?**

The Low Vision Working Group of VISION 2020 has endorsed a Standard List for low vision services.^1^ However, it may not always be possible to purchase all the items on the Standard List.

We have put together a list of the **minimum** equipment and devices you would need to offer a basic low vision service at district level. This list is based on our experience in the field, and we hope it will help you to start providing low vision support where no other service is available.

Keep accurate records of who you see and how they have been helped. Collect quotes from patients saying how they have benefited, and use these and your records to ask for further training, increased funding, and better equipment for your low vision clinic. Always refer people with complex needs for services at a higher level.

**Ophthalmic equipment**

- Streak retinoscope
- Direct ophthalmoscope
- An ordinary trial lens set; a full aperture trial set is preferable
- Universal trial frames
- At least one pair of paediatric trial frames
- Pen torch and measuring tape.

**Vision assessment equipment**

- Distant LogMAR test charts: at least have tumbling Es
- Near vision tests: at least have tumbling Es
- Reading acuity test. This can be created on computer using N or M sizes.

**Optical low vision devices**

- Spectacle magnifiers: locally made high positive add spectacles, from +4D to +12D, in steps of 2D.
- Four hand-held magnifiers (non-illuminated) from 5D to 20D. For example, one of 6D, one of 10D, one of 15D, and one of 20D
- Non-illuminated stand magnifiers from 10D to 25D. For example, one of 12D, one of 16D, one of 24D
- Use a variety of locally available sunglasses in different shades if filters are not available

**Non-optical devices**

- Reading/writing stand: locally made
- Reading slit, signature guide, and writing guide: all locally produced.

## Notes

**Both available on the Community Eye Health Update CD and online: www.cehjournal.org/journal.html**
